# Clinical significance of RNA methylation in hepatocellular carcinoma

**DOI:** 10.1186/s12964-024-01595-w

**Published:** 2024-04-02

**Authors:** Qiongling Bao, Yifan Zeng, Qizhuo Lou, Xuewen Feng, Shuwen Jiang, Juan Lu, Bing Ruan

**Affiliations:** State Key Laboratory for Diagnosis and Treatment of Infectious Diseases, National Clinical Research Center for Infectious Diseases, Collaborative Innovation Center for Diagnosis and Treatment of Infectious Diseases, The First Affiliated Hospital, National Medical Center for Infectious Diseases, Zhejiang University School of Medicine, No. 79 Qingchun Road, Shangcheng District, Hangzhou, Zhejiang 310003 China

**Keywords:** RNA methylation, Hepatocellular carcinoma, Expression features, Prognosis, Targeted treatments

## Abstract

Hepatocellular carcinoma (HCC) is a primary liver malignancy with high mortality rates and poor prognosis. Recent advances in high-throughput sequencing and bioinformatic technologies have greatly enhanced the understanding of the genetic and epigenetic changes in liver cancer. Among these changes, RNA methylation, the most prevalent internal RNA modification, has emerged as a significant contributor of the development and progression of HCC. Growing evidence has reported significantly abnormal levels of RNA methylation and dysregulation of RNA-methylation-related enzymes in HCC tissues and cell lines. These alterations in RNA methylation play a crucial role in the regulation of various genes and signaling pathways involved in HCC, thereby promoting tumor progression. Understanding the pathogenesis of RNA methylation in HCC would help in developing prognostic biomarkers and targeted therapies for HCC. Targeting RNA-methylation-related molecules has shown promising potential in the management of HCC, in terms of developing novel prognostic biomarkers and therapies for HCC. Exploring the clinical application of targeted RNA methylation may provide new insights and approaches for the management of HCC. Further research in this field is warranted to fully understand the functional roles and underlying mechanisms of RNA methylation in HCC. In this review, we described the multifaceted functional roles and potential mechanisms of RNA methylation in HCC. Moreover, the prospects of clinical application of targeted RNA methylation for HCC management are discussed, which may provide the basis for subsequent in-depth research on RNA methylation in HCC.

## Introduction

Primary liver cancer, predominantly hepatocellular carcinoma (HCC), is a significant global health concern because of poor 5-year average survival and high recurrence rates [1–6]. HCC has the sixth highest incidence rate among all cancers worldwide, with over half of the new annual cases and deaths occurring in China [2, 7, 8]. In terms of the etiology, HCC is multifactorial, and the main etiology includes chronic infections of hepatitis B and C viruses, alcohol consumption, metabolic liver disease, and ingestion of dietary aflatoxin [8–12]. With the rapid development of high-throughput sequencing technology in last decades, epigenetic modification is reported to be strongly associated with HCC progression [13–17]. Several reports revealed the abnormal epigenetic processes in HCC, such as histone alterations, DNA methylation, and chromatin remodeling [18, 19]. The intricate interplay between these genetic polymorphisms and environmental risk factors contributes to the complexity of HCC pathogenesis [20–22]. Current treatment options for HCC range from surgical treatment (liver resection and transplantation) and locoregional therapies such as chemoembolization and radiofrequency ablation to systemic therapies including targeted therapy (e.g., sorafenib and lenvatinib) and immunotherapy [23–27]. Although advances in the treatment methods for HCC have improved overall survival (OS), the therapeutic effects of currently available treatments are attenuated over time as disease progresses. Given the high heterogeneity, recurrence, and treatment resistance, dynamic regimens based on the patient’s pathogenesis mechanism and individual risk are becoming imperative [28]. An in-depth epigenetic analysis for HCC could provide unique insights into the pathogenetic mechanisms and molecule-targeted therapies in HCC [29–32].

Recent improvements in high-throughput sequencing and bioinformatic technologies have accelerated the understanding of genetic and epigenetic alterations in disease pathogenesis [33–37]. Epigenetic modifications refer to the post-translational modifications of heritable genes without DNA sequence alterations and primarily involve a group of histone and nucleic acid modifications [38, 39]. Among them, RNA methylation contributes to more than 60% of all types of RNA modifications. RNA methylation modifications mainly include N6-methyladenosine (m6A), 5-methylcytosine (m5C), N1-methyladenosine (m1A), N7-methylguanosine (m7G), and pseudouridine (Ψ) [40–42]. RNA methylation is observed to exist in all types of RNA, such as messenger RNA (mRNA), transfer RNA (tRNA), and noncoding RNA (ncRNA) [43–46]. These modifications involve regulating various aspects of targeted RNA processing, including RNA transcriptome processing, splicing, and export [47, 48]. It is acknowledged that m6A modification is the most abundant RNA modification in mammalian eukaryotic cells. It participates in several crucial physiological or pathological processes, such as cell proliferation, embryonic development, tumorigenesis, and neurogenesis [49–52]. RNA modification is a reversible process dynamically coordinated by a series of catalytically active methyltransferases, demethylases, and methylation-recognition proteins [53–55]. Taking the particularly well-investigated m6A methylation process as an example, adenylate methylation is performed by the methyltransferase complex. This complex is mainly composed of m6A writers such as methyltransferase, 3/14 (METTL3/14), WT1-associated protein (WTAP), and VIRMA (KIAA1429) [56]. Conversely, m6A demethylases α-ketoglutarate-dependent dioxygenase alkB homolog 5 (ALKBH5) and fat mass and obesity-associated protein (FTO) act as erasers to implement m6A demethylation. Importantly, m6A primarily occurs in the specific RRACH (R represents A or G and H represents A, C, or U) motif of adenine. Therefore, m6A binding proteins, including the YTH family, IGF2BPs, and HNRNP family, are required to specifically recognize m6A-modified bases and to drive downstream biological functions. Considering the potential reversibility of these changes, RNA methylation ensures its flexible response to external stimuli by altering gene expression and related functions, highlighting its valuable role in cancer therapy [57–59]. Increasing evidence indicates that RNA methylation plays a crucial role in tumorigenesis, and dysregulation of RNA methylation has been observed in various cancers including HCC [60–64]. Elucidating the multifaceted mechanisms of RNA modifications in the genetic pathogenesis of HCC is increasingly getting considered as a valuable direction for the clinical management of HCC [40, 65, 66].

In this review, we aimed to discuss the comprehensive understanding of RNA methylation in HCC based on the latest published findings. This review focused on the RNA methylation level, its functions, and main mechanisms related to HCC pathogenesis. In addition to the molecular mechanisms, we described the potential clinical application value of RNA methylation in HCC from prognostic and therapeutic viewpoints.

### RNA methylation in HCC

**Fig. 1:**
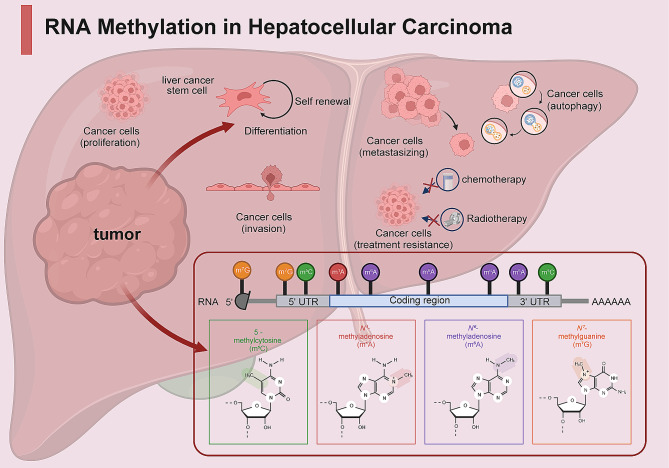
Landscape of RNA methylation regulators in HCC and their involvement in cellular processes. Various types of RNA methylation are observed to be dysregulated in the pathogenesis of HCC, including m6A, m5C, m1A, and m7G. Specifically, abnormal regulation of multiple RNA methylation processes involves the regulation of malignant cellular processes, such as cell proliferation, invasion, migration, treatment resistance, and even maintenance of stem cell properties

It has been widely reported that genetic and epigenetic alterations are accompanied by carcinogenesis and progression of HCC [67]. Considerable efforts have been directed toward extensively exploring genetic alterations and DNA methylation in HCC. A recent study highlighted the importance of RNA methylation in the etiology of HCC [68]. Many studies have reported significantly abnormal levels of RNA methylation in HCC cell lines and tissue samples compared with paracancerous tissues. Furthermore, abnormal expression of enzymes associated with RNA methylation (such as m6A methyltransferase METTL3 and demethylase ALKBH5, m7G methyltransferase METTL1, and m5C methyltransferase NSUN2) has been observed in liver cancer tissues (Table 1). This emphasizes the crucial functions of these enzymes in HCC pathogenesis and their promising diagnosis value (Fig. 1) [69–72]. Several studies have demonstrated that altered RNA methylation patterns are strongly associated with clinical pathological features of patients with HCC, mainly including tumor stage, distant metastasis, and poor prognosis [69, 73–75]. This association was evaluated to provide significant guidance for predicting the outcome of HCC. Integrated in-depth exploration of the mechanism of RNA methylation revealed that aberrant RNA methylation affects the activation of diverse genes and signaling pathways initiated in HCC carcinogenesis, including c-Myc, EGFR, WNT/β-catenin, and ETS1 [75–79]. By modulating the expression of cancer-related genes, RNA methylation drives the regulation of a wide range of processes in HCC progression, such as cell proliferation, invasion, resistance to therapy, tumor growth, and metastasis. Several studies have explored the interaction between RNA methylation and HCC progression and confirmed the great potential of targeted RNA methylation in vitro and in animal models (Table 2) [71, 73, 79, 80]. Thus, a thorough understanding of molecular pathology of RNA methylation in HCC is essential for the improvement of treatment landscape and clinical outcomes in HCC.

**Table 1:** Roles and mechanisms of RNA methylation in HCC

Type	Regulator of RNA methylation	Expression	Functions in HCC	Related targets	Underlying mechanisms	Year	Reference
Writer	METTL3	Upregulation	Promotes cell proliferation, migration, and lung metastasis	SOCS2	/	2018	[89]
Writer	METTL3	Upregulation	Promotes cell proliferation and inhibits cell apoptosis	EGFR	/	2023	[79]
Writer	METTL3	Upregulation	Promotes cell proliferation, migration, and invasion	miR6079	miR24-2, H3K9me3, and Pim1	2020	[81]
Writer	METTL3	Upregulation	Promotes cell viability, migration, and invasion	miR-589-5p	/	2022	[82]
Writer	METTL3	Upregulation	Promotes expansion, self-renewal, tumorigenicity, metastasis, and lenvatinib resistance of liver CSCs	FZD10	WNT/β-catenin and Hippo signaling pathways	2023	[69]
Writer	METTL3	Downregulation	Inhibits cell autophagy and sorafenib resistance	FOXO3	/	2020	[75]
Writer	KIAA1429	Upregulation	Promotes cell proliferation and motility	circDLC1	MMP1	2021	[90]
Writer	KIAA1429	Upregulation	Promotes cell proliferation and metastasis	GATA3	/	2019	[83]
Writer	WTAP	Upregulation	Promotes cell proliferation, cell cycle, and migration	ETS1	/	2019	[78]
Writer	ZCCHC4	Upregulation	Promotes cell proliferation	28SrRNA	/	2019	[84]
Eraser	ALKBH5	Upregulation	Promotes cell proliferation, migration, and invasion	circCPSF6	PCBP2 and YAP1	2022	[70]
Eraser	ALKBH5	Upregulation	Inhibits cell apoptosis and radiosensitivity, promotes cell proliferation	TIRAP	/	2023	[71]
Eraser	ALKBH5	Upregulation	Inhibits cell proliferation, migration, and invasion	LINC02551	DDX24	2022	[72]
Eraser	FTO	Downregulation	Inhibits cell proliferation and metastasis	CircGPR137B	MiR-4739	2022	[85]
Reader	YTHDF2	Upregulation	Promotes liver CSC, and lung metastasis	OCT4	/	2020	[88]
Reader	IGF2BP1	Upregulation	Promotes expansion and lenvatinib resistance of liver CSCs	MGAT5	/	2021	[91]
Writer	NSUN2	Upregulation	Promotes cell cycle, and sorafenib resistance	/	/	2023	[80]
Writer	NOP2	Upregulation	Promotes cell cycle, aerobic glycolysis, and proliferation	c-Myc	MAZ	2023	[76]
Writer	NSUN2	Upregulation	Promotes cell proliferation, migration, and invasion	H19lncRNA	G3BP1	2020	[96]
Writer	METTL1	Upregulation	Promotes cell radiotherapy resistance	tRNA	/	2023	[97]
Writer	METTL1	Upregulation	Promotes cell proliferation, migration, and invasion	SLUG/SNAIL	/	2023	[98]
Writer	METTL1	Upregulation	Promotes cell proliferation, migration, invasion, and lenvatinib resistance	EGFR	/	2023	[77]
Writer	METTL1	Upregulation	Promotes cell proliferation, migration, and invasion	/	/	2021	[108]
Writer	TRMT6/TRMT61A complex	Upregulation	Promotes cell cholesterol metabolism	/	/	2021	[73]

**Table 2:** Clinical applications of RNA modification in HCC

XXX	Regulator Type	Abnormal change	Clinical implication	Clinical data	Regulator of RNA methylation	Year	Reference
m6A	Methyltransferase	Upregulation	Prognosis	/	METTL3	2023	[69]
m6A	Methyltransferase	Downregulation	Prognosis and Treatment	HCC tumors (*n* = 3) and sorafenib-resistant tumors (*n* = 3) obtained at the Third Affiliated Hospital of Sun Yat‐Sen University and Southern Medical University	METTL3	2020	[75]
m6A	Methyltransferase	Upregulation	Prognosis	/	METTL3	2022	[82]
m6A	Methyltransferase	Upregulation	Treatment	HCC patients (*n* = 74) treated with adjuvant lenvatinib after radical treatment at the First Affiliated Hospital of Sun Yat-sen University	METTL3	2023	[79]
m6A	Methyltransferase	Upregulation	Diagnosis and Prognosis	/	KIAA1429	2019	[83]
m6A	Methyltransferase	Upregulation	Prognosis	/	WTAP	2019	[78]
m6A	Demethylase	Upregulation	Treatment	HCC samples (*n* = 17) collected from patients who received radiotherapy for intrahepatic tumor at the Nanfang Hospital of Southern Medical University	ALKBH5	2023	[71]
m6A	Demethylase	Downregulation	Prognosis	/	FTO	2022	[85]
m6A	Binding proteins	Upregulation	Prognosis	/	IGF2BP1	2021	[91]
m6A	Binding proteins	Upregulation	Prognosis	/	YTHDF2	2020	[88]
m6A	/	Upregulation	Treatment	Paraffin-embedded HCC tissue samples (*n* = 212) and fresh HCC tissue samples (*n* = 12) obtained from the Shenzhen People’s Hospital	global m6A levels	2023	[116]
m6A	Binding proteins	Upregulation	Treatment	HCC samples from the Cancer Genome Atlas (TCGA) and the Genotype-Tissue Expression (GTEx) databases	IGF2BP1	2022	[120]
m6A	Methyltransferase	Upregulation	Treatment	HCC frozen tissues (*n* = 17) and plasma (*n* = 35) from Fujian Medical University Union Hospital	METTL14	2022	[121]
m6A	Demethylase	Upregulation	Treatment	HCC tissues (*n* = 38) obtained from Eastern Hepatobiliary Surgery Hospital (Shanghai, China) and Mengchao Hepatobiliary hospital of Fujian Medical University	ALKBH5		[122]
m5C	Methyltransferase	Methyltransferase	Treatment	HCC tissues (*n* = 20) and the adjacent tissues (*n* = 20) collected from the Biobank of the First Affiliated Hospital of Zhengzhou University	NSUN2	2023	[80]
m5C	Methyltransferase	Upregulation	Prognosis	/	NSUN2	2020	[96]
m5C	Methyltransferase	Upregulation	Prognosis	/	NSUN4	2022	[106]
m5C	Methyltransferase	Upregulation	Prognosis	/	NSUN5	2022	[105]
m5C	Methyltransferase	Upregulation	Prognosis and Treatment	HCC (*n* = 40) and paraneoplastic tissues (*n* = 40) obtained from the Wuhan University Central South Hospital and Qilu Hospital of Shandong University.	NOP2	2023	[76]
m5C	Methyltransferase	Upregulation	Prognosis	/	ALYREF	2021	[107]
m7G	Methyltransferase	Upregulation	Treatment	MHCC97H orthotopic xenografts with or without IR treatment (*n* = 5/group) obtained from the First Affiliated Hospital of Sun Yat-sen University	METTL1	2023	[97]
m7G	Methyltransferase	Upregulation	Treatment	HCC patients’ paraffin tissues (*n* = 14) collected in the First Affiliated Hospital of Sun Yat-sen University	METTL1	2023	[98]
m7G	Methyltransferase	Upregulation	Treatment	HCC tissues with lenvatinib-sensitive (*n* = 37) and -resistant HCC (*n* = 13) collected in the First Affiliated Hospital of Sun Yat-sen University	METTL1	2023	[77]
m7G	Methyltransferase	Upregulation	Prognosis	/	METTL1	2021	[108]
m1A	Methyltransferase	Upregulation	Prognosis and Treatment	HCC tumor tissues (*n* = 191) and paired peri-tumor tissues (*n* = 191) obtained from the PLA General Hospital	TRMT6/TRMT61A	2021	[73]
m1A	/	Upregulation	Prognosis	/	m1A-related genes	2022	[74]
m6A/m5C/m1A	/	Upregulation	Prognosis	/	m6A/m5C/m1A regulated genes	2022	[109]
m6A/m5C/m1A	/	Upregulation	Prognosis	/	m6A/m5C/m1A regulated genes	2023	[110]

### Regulatory roles and mechanisms of RNA methylation in HCC

**Fig. 2:**
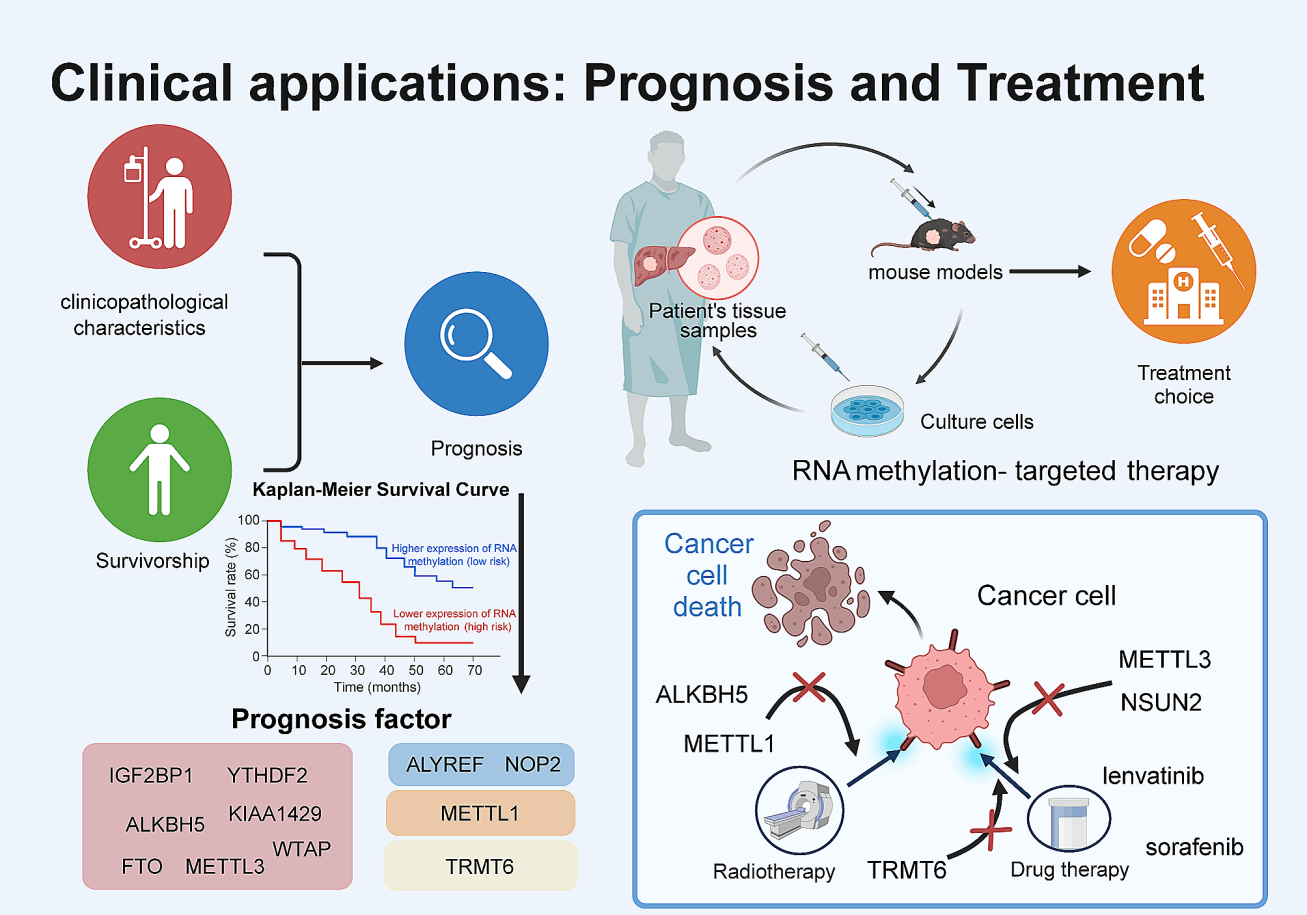
Dynamic processes of m6A modification and its underlying molecular mechanisms in HCC. Abnormal m6A prevalently exists in the most stages of HCC. m6A methylation is a dynamic modification mediated by m6A methylases, demethylases, and methylation-binding proteins. Abnormal expression of m6A regulators commonly affects downstream genes and signaling pathways, thereby contributing to the occurrence and development of HCC

Emerging evidence suggested a pivotal role of m6A modification in HCC progression (Fig. 2).

#### m6A methylation regulates RNA translation efficiency

Studies on mouse models with HCC reported that METTL3, by enhancing the translation of the epidermal growth factor receptor (EGFR) in lenvatinib-resistant HCC, promotes cell proliferation and inhibits apoptosis [79]. Furthermore, interactions between miRNAs and RNA-methylation-related genes are implicated in the regulation of HCC progression. For instance, increased expression of miR24-2 in HCC Hep3B cells is reported to indirectly elevate the levels of METTL3 and mature miR6079 through RNA methylation. Upregulation of miR6079 leads to increased histone H3 methylation (H3K9me3) and subsequent activation of the proto-oncogene Pim1, ultimately enhancing cell proliferation in HCC Hep3B cells [81]. In addition, METTL3 increases the expression of miR-589-5p and mediates the aggressive viability, migration, and invasion of Hep3B and SK-Hep1 cells [82]. As a noncanonical m6A methyltransferase, high expression of KIAA1429 in HCC SK-Hep1 and HCCLM3 cells mediates m6A methylation of the mRNA of GATA binding protein 3 (GATA3) precursor; this decreases the levels of GATA3 and promotes tumor growth and metastasis [83]. Moreover, WTAP, a key m6A methyltransferase, upregulates ETS proto-oncogene 1 (ETS1) by increasing ETS1 m6A modification in HCC Hep3B and HCCLM3 cells; this leads to a faster G2/M phase transition and HCC progression [78]. Additionally, CCHC zinc-finger-containing protein (ZCCHC4), a novel m6A methyltransferase, specifically targets human 28 S rRNA, altering translation activity and enhancing the proliferative ability of HCC HepG2 cells [84]. RNA immunoprecipitation analysis has revealed that the m6A methylation of circCPSF6, mediated by ALKBH5, promotes its interaction with poly(C)-binding protein 2 (PCBP2) to regulate Yes-associated protein 1(YAP1) expression, thereby driving the aggressive viability, migration, and invasion of HCC cells [70]. Moreover, ALKBH5 directly downregulates LINC02551, which slows down the growth and metastasis of HCC cells by weakening the stability of DEAD box protein 24 (DDX24) [72]. During radiation therapy for HCC, radiation-induced hepatic stellate cells upregulate ALKBH5 to mediate the methylation modification of tollinterleukin 1 receptor domain containing adaptor protein (TIRAP), resulting in reduced apoptosis and radiosensitivity of HCC cells [71]. In HCC HepG2 and Hep3B cells, circGPR137B has been identified as a sponge for miR-4739 and FTO as a direct target of miR-4739, forming a positive feedback loop. This circGPR137B/miR-4739/FTO axis inhibits cell proliferation, invasion, and lung metastasis [85]. Cancer stem cells (CSCs) are a subpopulation of tumor cells exhibiting self-renewal and multi-directional differentiation capabilities, which serve as the primary driving forces for tumor development and drug resistance [86, 87]. METTL3 has been reported to enhance the expression of Frizzled-10 (FZD10) by mediating m6A methylation of FZD10 mRNA, thereby promoting the expansion of liver CSCs in HCC through the activation of β-catenin and Hippo signaling pathways [69]. Several studies have reported the regulatory roles of YTHDF2 on liver CSCs. Elevated YTHDF2 expression has been observed to promote the protein expression of the pluripotency factor OCT4, leading to increased formation of cell spheres and subsequent lung metastasis [88].

#### m6A methylation regulates RNA structure and stability

Additionally, METTL3 augments the methylation of the mRNA of suppressor of cytokine signaling 2 (SOCS2) in HCC cells, facilitating the selective binding of YTHDF2 to the methylated mRNA of SOCS2 and its subsequent degradation in the cytoplasm. This collaborative action of METTL3 and YTHDF2 promotes the proliferation, migration, and colony formation of HCC cells [89]. Recent studies have highlighted the role of KIAA1429 in regulating circDLC1 expression; it is downregulated in HCC Huh7 and SKHep1 cells. Reduced circDLC1 impairs the stability and expression of matrix metallopeptidase 1 (MMP1), contributing to enhanced invasion and migration of HCC cells [90]. Under hypoxic conditions in HCC, downregulated METTL3 eliminates the stabilization of FOX3 mRNA through a YTHDF1-dependent mechanism, contributing to the activation of autophagy and sorafenib resistance in sorafenib-resistant HCC cells [75]. It has been suggested that the binding of IGF2BP1 to the target gene α-1,6-mannosyl protein 6-β-n-acetylglucosamine aminotransferase (MGAT5) affects the stability of the mRNA of MGAT5 and further elevates its expression. By upregulating MGAT5, IGF2BP1 promotes self-renewal and chemoresistance of liver CSCs and enhances the expression of stemness genes [91].

#### m5C modification regulates RNA stability

**Fig. 3:**
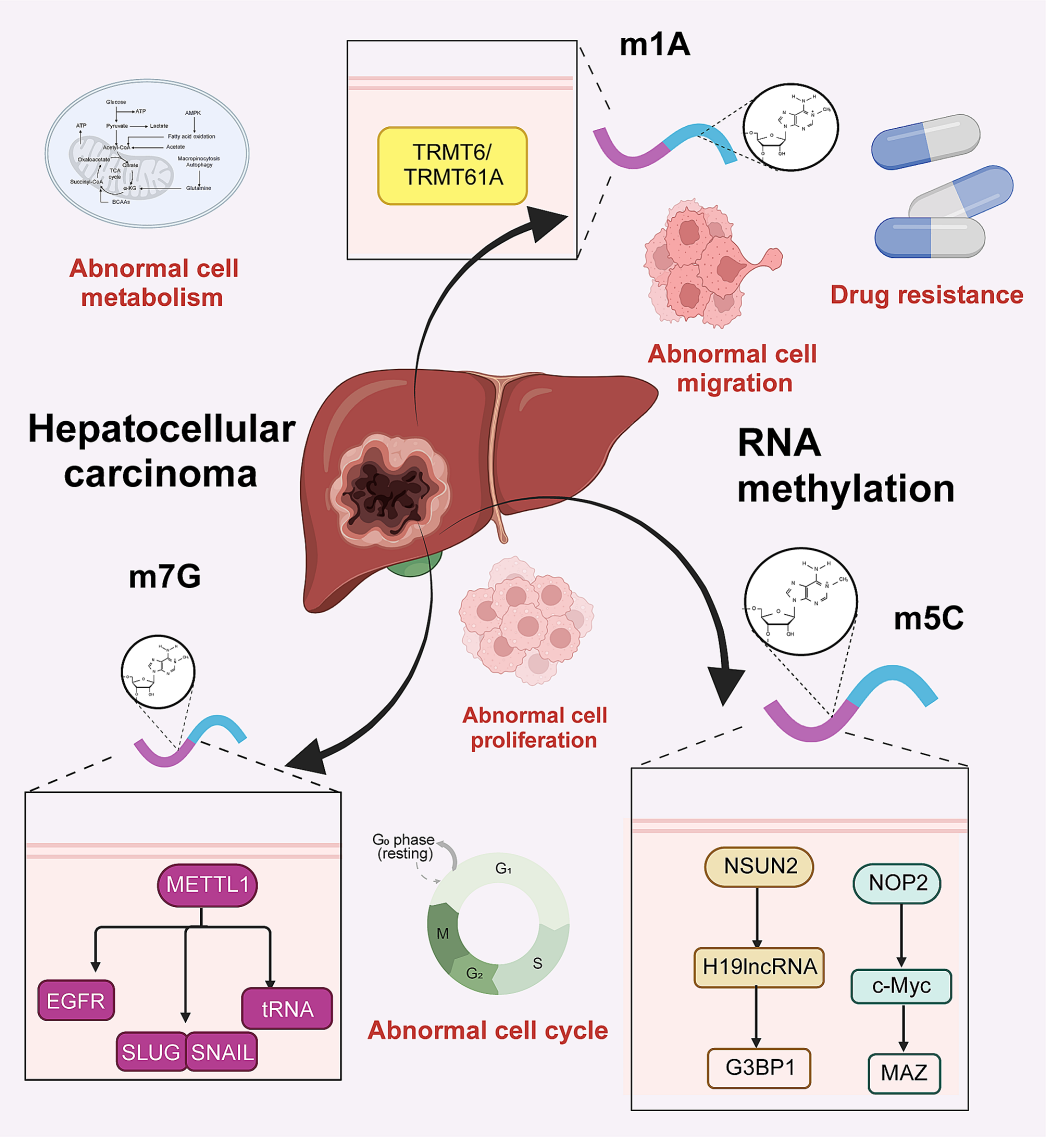
Molecular mechanisms and functions of m5C, m7G, and m1A modification in HCC. In addition to m6A modification, m1A, m7G, and m5C methylation also play important roles in the pathogenesis of liver cancer. m1A, m7G, and m5C methylation regulate the aberrant expression of corresponding RNAs, thereby participating in various malignant biological processes of liver cancer, such as cell proliferation, migration, drug resistance, abnormal metabolism, and disrupted cell cycle

Numerous studies revealed that the abnormal levels of m5C modification, a common crucial methylation, significantly contribute to the occurrence and progression of HCC (Fig. 3) [92–95]. Studies indicated that the overall level of m5C is substantially upregulated in HCC, and numerous mRNAs modified by m5C are involved in various oncogenic pathways [80]. The high expression of NSUN2 plays a critical role in mediating the carcinogenesis of m5C in HCC. By activating the Ras pathway, NSUN2 inhibits sensitivity of Huh7 cells to sorafenib, leading to impaired apoptosis rate and cell cycle arrest. In addition, NSUN2 has been demonstrated to mediate m5C modification of H19 long ncRNA (lncRNA), thereby enhancing the stability and expression of H19 lncRNA. Overexpressed H19 lncRNA specifically binds to Ras-GTPase-activating protein-binding protein 1 (G3BP1), thus promoting malignant behaviors such as proliferation, invasion, and metastasis of HCC HepG2 cells [96].

#### m5C modification regulates RNA translation efficiency

The m5C methyltransferase NOP2 is highly expressed in liver cancer. Overexpression of NOP2 magnifies the proliferative, invasive, and migratory capacities of HCC Hep-3B cells. Mechanistic investigations have demonstrated that NOP2 positively regulates the expression of transcription factor c-Myc through m5C modification, thereby promoting aerobic glycolysis. The upstream MYC-associated zinc finger protein (MAZ) was further confirmed to directly bind to the promoter region NOP2 and to promote its transcription. Therefore, the MAZ/NOP2/c-Myc axis has a crucial effect in metabolic reprogramming and progression of liver cancer [76].

#### m7G and m1A regulate RNA translation efficiency

As a crucial m7G methyltransferase, METTL1 also mediates the translation of tRNAs related to DNA repair, thereby functioning as an essential enhancer in HCC cell proliferation and radioresistance [97]. In the condition of sublethal heat exposure after insufficient radiofrequency ablation, METTL1 was observed to facilitate the translation of transcription factor SLUG/SNAIL via m7G tRNA modification, exerting enhanced malignant capabilities in HCC SNU449 and MHCC97H cells [98]. An unbiased proteomic profiling conducted on the parental and lenvatinib-resistant HCC cells revealed the significant upregulation of two key components of m7G methyltransferase complex, including METTL1 and WD repeat domain 4 protein (WDR4). A series of in vitro and in vivo experiments validated that overexpressed METTL1 is involved in lenvatinib resistance and promotion of proliferation capacity of lenvatinib-resistant HCC cells by elevating the translation of EGFR [77]. m1A modification mediated by the TRMT6/TRMT61A complex enhances the translation of peroxisome proliferator-activated receptors (PPARs), which in turn activate the Hedgehog signaling and promote cholesterol biosynthesis of liver CSCs. Studies have reported PPARδ-induced accelerated self-renewal of liver CSCs and tumor growth promoted by enhanced cholesterol biogenesis [73].

### Clinical applications of RNA modification in HCC

#### Diagnostic and prognostic value of RNA modification in HCC

**Fig. 4:**
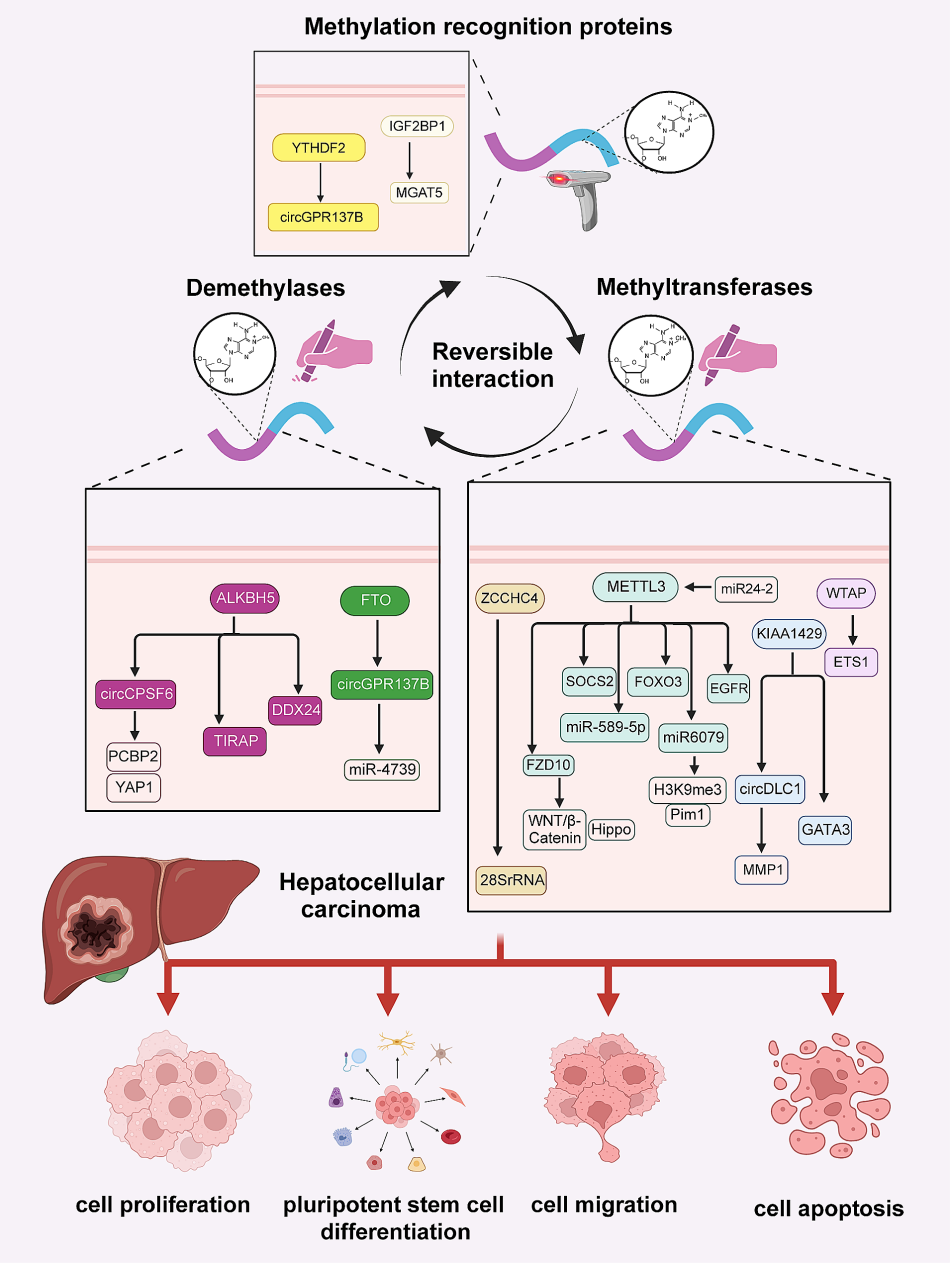
Promising clinical applications of RNA methylation in HCC

In recent years, numerous studies have highlighted the crucial role of RNA methylation in the pathogenesis of liver diseases, demonstrating their significant clinical potential in liver cancer, particularly in HCC [99, 100]. Abnormal expression of RNA-methylation-related genes, including regulators and target genes, has been widely reported to be associated with survival and clinical features. This suggested that these genes may serve as diagnostic and prognostic indicators for patient survival (Fig. 4) [101–104].

For m6A modification, METTL3 overexpression is associated with poor prognosis in HCC, along with larger tumor size, worse Edmondson–Steiner grade, and advanced tumor stage [82, 89]. In addition, the diagnostic value of KIAA1429 level has been confirmed through receiver operating characteristic curve analysis in multiple HCC cohorts [83]. Elevated KIAA1429 expression is associated with unfavorable OS and disease-free survival rates in patients with HCC. WTAP expression is closely associated with clinical pathological features such as tumor encapsulation and recurrence in HCC. High expression of WTAP is correlated with poorer OS and disease-free survival rates, making it an independent prognostic factor in patients with HCC. The coexpression of WTAP and ETS1 has been reported to significantly impact patient outcomes in HCC. High levels of IGF2BP1 are correlated with larger tumor size, lymph node metastasis, and poorer survival rates in patients with HCC [91]. YTHDF2 expression is negatively correlated with survival rates in patients with HCC, further highlighting its potential as a prognostic marker [88]. In addition, the elevated expression of FZD10, METTL3, and YTHDF2 has been linked to poorer prognosis in patients with HCC, leading to worse OS and disease-free survival rates. The combination of these three markers may have more accurate prognostic predictive value [69]. Reduced FTO expression is reported to be associated with older age and distant metastasis in patients with HCC. However, high FTO expression is associated with better OS rates [85].

In addition to m6A, other RNA-modification-related genes have been investigated for their diagnostic and prognostic values in HCC. In terms of m5C modification, NOP2 has been reported to be highly expressed in HCC and is associated with advanced tumor characteristics, including size, staging, grading, and portal vein tumor thrombosis. Increased NOP2 expression is correlated with decreased OS in patients with HCC and is identified as independent prognosis-predicting factor in HCC [76]. Moreover, the expression levels of NSUN2, NSUN4, and NSUN5 are closely related to the clinical features of HCC and are associated with advanced tumor characteristics and poorer prognosis [76, 96, 105, 106]. Overexpression of ALYREF, another m5C methyltransferase, is linked to more aggressive tumor grading and unfavorable prognosis in patients with HCC [107]. Abnormal elevation in m1A levels is observed in liver CSCs and HCC tumor tissues, particularly in poorly differentiated samples with microvascular invasion [73, 74]. Targeting m1A-related genes could serve as a therapeutic strategy for managing HCC. For m7G modification, high expression of METTL1 or WDR4 has been confirmed to be associated with advanced tumor stage, vascular invasion, and poor survival in patients with HCC using TCGA LIHC dataset [108]. Moreover, prognostic risk models based on the expression of m6A/m5C/m1A-regulated genes in HCC tissue have exhibited promising results in accurately reflecting poor prognosis for patients with HCC [109, 110].

Although RNA modification holds promising prospects for liver cancer diagnosis and prognosis, significant challenges still need to be addressed. Currently, the sensitivity and specificity of RNA modification-based biomarkers have not been clearly elucidated. Future efforts will require extensive clinical research to develop and validate the sensitivity and specificity of methylation-specific biomarkers. Overall, these findings highlight the diagnostic and prognostic potential of RNA modification in HCC and suggest that targeting RNA-methylation-related genes could be a promising approach for HCC management.

### Therapeutic potentials of RNA modification in HCC

Growing evidence has supported the clinical significance of RNA methylation in liver cancer, it is essential to delve deeper into ongoing research in RNA methylation-based drug discovery. Previous studies have highlighted the close relationship between autophagy and chemotherapy resistance in tumor cells. Autophagy is induced during chemotherapy to evade cell death, leading to chemotherapy resistance [111–115]. Recent studies have shed light on the role of m6A methylation in regulating tumor autophagy and chemotherapy resistance. For instance, in an orthotopic liver tumor model, the ablation of the METTL3/FOXO3 axis was reported to weaken the antitumor efficacy of sorafenib, leading to enhanced tumor autophagy, angiogenesis, and a subsequent acceleration of tumor growth [75]. Furthermore, promising strategies such as inhibiting METTL3 with the compound STM2457 have shown potential in enhancing the sensitivity of lenvatinib in preclinical settings [79]. In patients with HCC resistant to radiation therapy, ALKBH5 upregulation serves as a marker for evaluating the efficacy of radiotherapy [71]. Additionally, AC115619, a liver-specific lncRNA encoding a micropeptide AC115619-22aa, reduces m6A levels. This can be a novel approach to inhibit HCC progression in animal and patient-derived models [116].

Furthermore, exploring the therapeutic potential of m5C modification can broaden treatment options for liver cancer. Studies have highlighted the role of NSUN2 in exacerbating sorafenib resistance and chemotherapy efficacy by promoting Ras pathway activation in HCC cells [80]. Insights from patient-derived xenograft models suggest the beneficial impact of *NOP2* gene silencing on the growth of HCC tumors, especially when combined with sorafenib therapy [76]. The regulatory role of METTL1 in HCC radioresistance and DNA repair highlights its importance in HCC radiation therapy [97]. Targeting the METTL1-SLUG/SNAIL axis shows promise in mitigating heat stress responses and reducing HCC recurrence after radiofrequency ablation [98]. Additionally, the inhibitor thiram, targeting the TRMT6/TRMT61A complex, has demonstrated efficacy in suppressing the growth of established HCC mouse xenograft models and inhibiting the formation of tumor spheroids in HCC cell lines, emphasizing the pivotal role of m1A methylation in the prognosis and therapy of HCC [73].

In recent years, significant progress has been made in tumor immunotherapy, particularly in the utilization of immune checkpoint inhibitors (ICIs), offering new therapeutic prospects for patients with advanced liver cancer [117–119]. The role of RNA modification in ICIs has emerged as a compelling area of interest in liver cancer. Recent studies have revealed that RNA methylation plays a pivotal role in regulating the status and function of immune cells within the immune microenvironment, as well as the interaction between hepatoma tumor cells and immune cells in liver cancer through the modification on related genes and signaling pathways. RNA methylation can impact the expression levels of ICIs, such as PD-1, PD-L1, CTLA4, and other checkpoints, thereby influencing the efficacy of ICIs. For example, research has highlighted that the small molecule cucurbitacin B (CuB) can effectively target IGF2BP1, inhibit the recognition of c-MYC by IGF2BP1, induce apoptosis, potentially recruit immune cells to the tumor microenvironment, and suppress the expression of PD-L1, thereby demonstrating anti-HCC effects [120]. Lipopolysaccharide (LPS) also plays a significant regulatory role in the expression of PD-L1 in liver cancer cells. LPS upregulates METTL14 to promote m6A methylation of MIR155HG, with dependence on the “reader” protein ELAVL1 (also known as HuR) to stabilize MIR155HG. Subsequently, MIR155HG acts as a competitive endogenous RNA to modulate the expression of PD-L1 through the miR-223/STAT1 axis, thereby enhancing immune evasion in HCC [121]. Moreover, sequencing of the m6A methylome has also unscored that PD-L1 mRNA is a direct target of m6A modification, with its levels being regulated by ALKBH5. In intrahepatic cholangiocarcinoma, ALKBH5 suppresses the expansion and cytotoxicity of T cells by maintaining the expression of PD-L1 on tumor cells. These findings shed light on the potential role of ALKBH5 in immune therapy response [122]. However, the current research focusing on RNA methylation to improve the efficacy of immunotherapy in liver cancer is primarily based on animal models and in vitro experiments, with limited clinical application. Although promising results have been obtained in laboratory settings and related studies suggest a potential role for RNA modification in enhancing immunotherapy, the translation of these research findings into clinical practice still faces challenges [123–126]. In clinical research, more clinical trials and investigations are needed to assess the safety and efficacy of RNA methylation in liver cancer immunotherapy. A more in-depth understanding of the oncogenic mechanisms of RNA methylation in liver cancer, as well as the translation of RNA methylation modifications into clinical practice, is necessary to maximize the benefits of immunotherapy for liver cancer patients. Collectively, these results support that RNA modification can regulate mRNA levels of immune checkpoint molecules such as PD-L1, thereby influencing the response of tumor to immunotherapy. RNA modification may serve as a novel pharmacological target for improving low responsiveness of immunotherapy.

Despite the optimistic outlook on RNA methylation in liver cancer treatment, there are notable challenges that need to be addressed. These challenges include gaining a comprehensive understanding of the complex interactions between RNA methylation and cellular processes, as well as overcoming obstacles related to drug delivery. Additionally, developing RNA methylation enzyme inhibitors or activators with high specificity and low toxicity, as well as precisely regulating RNA methylation levels to mitigate potential side effects represent significant hurdles. Furthermore, the role of RNA methylation in different tumor microenvironments may vary, requiring more detailed molecular mechanism studies. Therefore, further exploration and detailed discussion of ongoing research in RNA methylation-based drug discovery are warranted to advance the field. This will facilitate the translation of basic findings of RNA methylation into effective clinical treatment strategies s, ultimately enhancing patient outcomes in liver cancer treatment. Overall, these findings highlight the potential of RNA-modification-targeting strategies to enhance the treatment against HCC, offering novel avenues for improving patient outcomes in this challenging cancer landscape. With continuous advancements in technologies, it is hoped that there will be more clinical studies on the effects of RNA methylation in liver cancer immunotherapy, providing additional support and guidance for the development of more effective treatment strategies.

## Conclusion

Advances in high-throughput sequencing technologies have revealed a complex map of RNA methylation in the context of HCC and have explored its close association with HCC progression. Aberrant RNA methylation alterations, specifically m6A, are implicated in the pathogenesis of HCC. As a crucial post-transcriptional modification, dysregulation of RNA methylation and related writers, erasers, and readers has been observed in HCC. This can lead to altered patterns of gene expression and certain signaling pathways. These changes contribute to HCC initiation and progression by mediating diverse cellular processes, mainly including cell proliferation, invasion, treatment resistance, and metastasis. In addition to understanding the molecular mechanisms, RNA methylation has great clinical application of in HCC. The aberrant expression of key enzymes related to RNA methylation is associated with clinicopathological features and patient prognosis in HCC. Furthermore, multiple prognostic models based on the expression of RNA-methylation-related genes have been developed, have demonstrated strong predictive performance, and have proved to be promising for predicting clinical outcomes in HCC. Further external validation is needed to confirm their reliability and broad applicability in the management of HCC prognosis. In addition, epigenomic-targeted therapies may provide more combination strategies for treating HCC. Targeted RNA methylation has been well-tested alone or in combination with chemotherapies or radiotherapies for treating HCC in diverse animal trials. However, importantly, approved targeted RNA-modification-related drugs are currently lacking in HCC. These studies are still in the early stages and further large clinical validation is necessary to determine the practical application of RNA-methylation-targeted treatment in HCC.

The expression of RNA-methylation-related regulators is closely correlated with the malignant clinicopathological features of patients with HCC. Diverse prognostic models based on RNA methylation regulators are validated to be effective in predicting the prognosis of patients with HCC in several online datasets. In addition, RNA-methylation-targeted therapy has exhibited efficacy in numerous preclinical HCC models, exhibiting novel clinical applications in HCC.

## Data Availability

No datasets were generated or analysed during the current study.
